# Exploring the Feasibility of Direct-Dispersion Oleogels in Healthier Sausage Formulations

**DOI:** 10.3390/gels10120819

**Published:** 2024-12-12

**Authors:** Niaz Mahmud, Md. Jannatul Ferdaus, Roberta Claro da Silva

**Affiliations:** Food and Nutritional Sciences Program, North Carolina A&T State University, Greensboro, NC 27411, USA; nmahmud@aggies.ncat.edu (N.M.); mferdaus@aggies.ncat.edu (M.J.F.)

**Keywords:** oleogel, direct-dispersion, fat replacement, sausage, nutritional enhancement, sensory, texture, oxidative stability

## Abstract

Oleogels developed through the direct-dispersion method offer an innovative, scalable, and efficient alternative to traditional fats in sausage production, providing a solution to health concerns associated with the high saturated fat content of conventional formulations. By closely mimicking the texture, stability, and mouthfeel of animal fats, these oleogels provide a novel approach to improving the nutritional profile of sausages while maintaining desirable sensory characteristics. This review critically evaluates cutting-edge research on oleogels, emphasizing innovations in their ability to enhance emulsion stability, increase cooking yield, reduce processing weight loss, and optimize fatty acid composition by reducing overall fat and saturated fat levels. Despite their potential, sausage formulations with oleogel still face challenges in achieving consistent sensory properties, texture, and oxidative stability, often failing to fully replicate the sensory qualities and shelf-life of animal fats. To push the boundaries of oleogel technology and meet the increasing demand for healthier, high-quality sausage products, we propose focused innovations in refining oil-to-gelator ratios, exploring a wider range of novel gelators, optimizing production methods, and developing cost-effective, scalable strategies. These advancements hold significant potential for revolutionizing the sausage industry by improving both the technological and nutritional qualities of oleogels.

## 1. Introduction

With growing emphasis on nutritious eating habits, the food industry is challenged to create nutritious products that satisfy both health needs and consumer expectations for taste and texture [1,2]. Consumers today are more informed about the health risks associated with processed foods, particularly those high in saturated fats, sodium, and nitrates, which are commonly used in sausage production to enhance flavor, texture, and shelf-life [3,4]. Sausages, historically valued for their flavor and versatility, are traditionally produced with high levels of these ingredients, posing significant health risks such as cardiovascular disease and certain types of cancer [5]. In response, there has been a surge in innovation aimed at developing healthier alternatives that maintain product quality while meeting modern health standards. This shift has led to the exploration of various fat substitutes, among which oleogels have emerged as a promising option [6]. Due to their ability to mimic the texture and mouthfeel of animal fats while reducing saturated fat and cholesterol, oleogels offer unique advantages in sausage production by enabling a significant reduction in harmful fats without compromising taste and palate experience [7]. However, replicating the sensory qualities of traditional fats while reducing saturated fat content remains a formidable challenge, as achieving a comparable mouthfeel, emulsion stability, and flavor profile is critical to consumer acceptance.

This review begins by providing a brief overview of the historical development of sausages and an in-depth exploration of current market dynamics in sausage production, offering insights into the health implications associated with conventional ingredients and highlighting the growing trend toward healthier alternatives within the industry. With a particular emphasis on oleogels as an innovative solution, this analysis delves into their unique structuring mechanisms and evaluates their impacts on emulsion stability, cooking yield, and nutritional composition. Furthermore, it examines oleogels’ potential to enhance the textural and sensory qualities of sausage products while improving oxidative stability, thus offering a viable pathway toward reducing harmful fats without compromising consumer expectations. By synthesizing the latest research findings and identifying persistent challenges, this review aspires to inform future advancements in oleogel applications, ultimately contributing to the creation of nutritionally enhanced sausage products that align with contemporary consumer demands for health and quality.

## 2. Sausages—Brief History and Current Market Size

Sausages have long been a staple in global cuisine, known for their versatility and flavor in preserving and consuming meat. They consist of comminuted meat mixed with fat, salt, and seasonings, encased in natural or synthetic casings [5]. Dating back to around 1500 BC, sausages were consumed by Babylonians and ancient Chinese, with the term “sausage” derived from the Latin word salsus, meaning salted or preserved [8,9]. By 449 BC, the Greeks frequently mentioned salami, and early sausage-making focused on preserving meat through salting and drying. Dry sausages were common in warm Southern Europe due to the lack of refrigeration, while fresh sausages thrived in cooler Northern Europe [9]. By the mid-19th century, during the U.S. Civil War, the sausage industry expanded with advances in refrigeration and meatpacking. Immigrants brought their traditional recipes, contributing to a wide range of sausage types. In the early 1900s, unsafe and unsanitary practices in meat production, including sausages, were exposed by Upton Sinclair’s 1906 novel *The Jungle*. This public outcry led to the Federal Meat Inspection Act (FMIA), which required inspections to ensure cleanliness, accurate labeling, and transparency in meat products. The FMIA helped improve the safety and quality of sausages, ensuring they were made from safe ingredients, and laid the foundation for modern food safety standards. Today, stringent regulations continue to safeguard the production of diverse and safe sausage products worldwide [10].

The preparation methods, ingredients, and consumption of sausages vary greatly across regions, reflecting cultural preferences, local resources, and numerous variations in production styles. Sausages can be broadly classified into categories based on their processing methods—fresh, cured, cooked, smoked, and fermented—and this diversity makes them a versatile product suitable for everyday meals to fine dining creations [9]. The global sausage market is projected to generate $110.70 billion in revenue in 2024, with an annual growth rate of 5.28%. Germany leads in revenue generation, contributing $10.82 billion. By 2029, the global market volume is expected to reach 15.53 billion kg [11]. In the United States, the sausage market is expected to reach $6.47 billion in revenue in 2024, growing by 5.48% annually. The U.S. market volume is projected to reach 730 million kg by 2029, with a 3.9% growth in 2025 [12]. These figures indicate strong and steady demand for sausages globally driven by consistent consumption patterns and volume expansion.

## 3. Health Risks of Sausage Consumption from Saturated Fats, Sodium, and Nitrates

As sausage consumption increases, so do the associated health risks, largely driven by traditional production methods that depend heavily on saturated fats, high sodium levels, and nitrate preservatives. Sausages are traditionally formulated with fats such as lard and tallow, both of which contain significant proportions of saturated fats—approximately 40% in lard and 50% in tallow [13]. Scientific research consistently shows a strong correlation between high saturated fat intake and elevated Low-Density Lipoprotein (LDL) cholesterol, a leading contributor to atherosclerosis [14,15]. This condition results in fatty deposits building up in the arteries, elevating the risk of cardiovascular diseases, including heart attacks and strokes. Saturated fats have also been linked to chronic inflammation, which can lead to insulin resistance—a key factor in the development of type 2 diabetes [16].

In addition to their high saturated fat content, traditional sausages are typically rich in salt and nitrates. Salt enhances flavor and extends the product’s shelf-life; however, excessive consumption presents significant health risks. Research has consistently demonstrated that elevated salt intake is a major contributing factor to hypertension and is directly associated with an increased risk of cardiovascular diseases, including heart failure and stroke [17,18,19]. The American Heart Association recommends keeping sodium intake below 2300 milligrams daily, yet a single serving of sausage can contain up to half of this recommended daily limit, putting consumers at higher risk for sodium-related health issues [17,18,19,20]. Nitrates, commonly used to preserve and color sausages, can pose additional health risks. When exposed to high temperatures during cooking, nitrates can transform into nitrosamines, compounds classified as carcinogenic, particularly linked to an increased risk of colorectal cancer [21]. Research by the World Health Organization and various other studies in epidemiology has emphasized the cancer risk associated with high consumption of processed meats containing nitrates [22,23]. Frequent consumption of such processed meats, especially those rich in nitrosamines, has been shown to correlate with a higher incidence of digestive tract cancers, with colorectal cancer being the most prevalent among them [22,23]. With growing awareness of health risks, there is a clear need for changes in sausage production to promote healthier options. Reducing traditional saturated fats and minimizing sodium and nitrate-based preservatives can lower risks associated with heart disease, cancer, and hypertension [24]. Shifting to these healthier practices aligns with public health goals and meets consumer demand for better nutritional choices.

## 4. Current Market Trends in Healthier Sausage Production

Over recent years, sausage production has evolved with a growing emphasis on health, sustainability, and innovation. Consumers now have access to a variety of options, including low-fat, plant-based, and hybrid sausages, aimed at reducing fat content and catering to health-conscious individuals. Some of these alternatives are fortified with fiber and omega-3 fatty acids to boost their nutritional profiles [25,26,27,28,29,30,31]. In parallel, ethical sourcing and eco-friendly packaging have addressed sustainability concerns, while clean labels with simple, recognizable ingredients and global flavors have expanded the variety of offerings. Specialty options, such as gluten-free and keto-friendly sausages, further accommodate specific dietary needs.

The most significant trend in sausage products now is perhaps the growing demand for low-fat options, as consumers prioritize healthier choices in today’s world [32]. The low-fat sausage market is projected to grow at a 7.93% annual growth rate from 2023 to 2031, reaching a value of $102.37 billion. By 2024, it is expected to hit $60 billion, driven by technological advancements, rising consumer demand, and strategic investments by industries, setting the stage for sustained market growth [33]. Key players in the low-fat sausage industry are actively innovating to meet the growing consumer demand for healthier options. Atria Oyj, a Finnish food company, is focusing on creating healthier, leaner sausage products as part of its broader strategy to cater to health-conscious consumers. Beyond Meat (El Segundo, CA, USA) is leveraging its expertise in plant-based alternatives, offering low-fat sausages to meet the increasing demand for meat substitutes. Heck Food (Kirklington, Bedale, UK) specializes in high-protein, low-fat sausages made with chicken and plant-based ingredients. Hormel Foods (Austin, MN, USA) has also expanded its portfolio to include leaner sausage options, aligning with consumer health trends. Meanwhile, JBS S.A., (São Paulo, Brazil) one of the world’s largest meat processors, has introduced low-fat beef and pork sausages to reduce calorie content while maintaining flavor. Johnsonville (Sheboygan Falls, WI, USA) is focusing on reducing fat content in its traditional sausages to cater to health-conscious customers. Nestlé (Vevey, Switzerland), through its Garden Gourmet brand, is exploring plant-based sausage options that are low in fat. Premium Brands Holding Corporation (Richmond, ON, Canada) and Tyson Foods (Kernersville, NC, USA) are also innovating by using leaner cuts of meat and plant-based alternatives. Smithfield Foods (Smithfield, VA, USA) produces leaner pork sausages as part of its healthier product line [34,35]. 

While these healthier endeavors in sausage production have gained considerable traction, offering low-fat options and replacing saturated fats with healthier lipid sources remain challenging. The exploration of alternative lipids and structuring them to mimic traditional fat sources is still in its early stages. Research into these substitutes aims to maintain the texture, flavor, and mouthfeel that consumers expect from conventional sausages while reducing fat content. As such, advancements in this area are ongoing, with many producers awaiting scalable and economically viable solutions to incorporate healthier lipid replacements effectively.

## 5. Challenges in Low-Fat and Reduced-Saturated-Fat Sausage: Oleogel as a Promising Solution

Despite advancements in low-fat sausage production, the primary challenge remains replicating the flavor, texture, and emulsion stability provided by traditional fats [6,36,37,38,39]. Traditional fats are integral to the sensory qualities that define sausages, making it difficult to replace them without compromising taste and mouthfeel. While plant-based and modified products offer lower-fat alternatives, they often fall short of matching the rich texture and binding properties consumers expect [40]. A recent survey indicated that while 36% of consumers actively seek low-fat meat options, nearly 38% would still opt for higher-fat versions when given the choice, reflecting a notable resistance to low-fat products. This reluctance is primarily driven by concerns over taste, texture, and cultural attachment to traditional meat products [41]. In response, research efforts have increasingly focused on identifying lipid sources that reduce both saturated and overall fat content in food products without compromising quality. Among these, oleogels have emerged as a leading innovation [42]. Oleogels are lipid-based materials that convert liquid oils into gel-like structures. They are composed of three key components: the oil phase, a gelator, and optional additives [7]. The oil phase, typically making up 80–99% of the oleogel, includes vegetable oils (e.g., sunflower oil, canola oil, soybean oil, olive oil), providing the liquid base for gelation. The gelator, which constitutes 1–20%, is the essential component that forms the gel network [43]. Gelators can be low molecular weight compounds (LMWGs) or polymers. Low molecular weight gelators include waxes (e.g., beeswax, carnauba wax), fatty acids (e.g., stearic acid), and mono- and diglycerides, which form a gel by crystallizing or self-assembling in the oil. High-molecular-weight gelators (HMWGs) (e.g., ethyl cellulose, proteins, polysaccharides, starches) form gels through polymer entanglement, cross-linking, microscopic phase separation, conformational transition, or mesophase formation [6,44]. Optional additives, such as emulsifiers (e.g., lecithin), antioxidants (e.g., tocopherols), and flavors, can be incorporated to enhance the texture, shelf-life, or sensory qualities of the oleogel emulsifiers stabilize the system by reducing interfacial tension between components and improving the dispersion of gelators within the oil phase. Additionally, they reinforce the gel network, preventing phase separation and enhancing the structural integrity and thermal stability of the oleogel [45,46]. Antioxidants, on the other hand, protect the oil from oxidation by neutralizing free radicals and reactive oxygen species, which can initiate or propagate oxidative reactions [47,48,49]. By preventing the breakdown of unsaturated lipids, antioxidants further contribute to the stabilization and preservation of the oleogel [50,51].

Oleogels stabilize liquid oils within a gel matrix, effectively mimicking the properties of solid fats. This allows manufacturers to significantly reduce saturated fat and cholesterol levels while preserving essential sensory qualities, such as mouthfeel and texture, to meet consumer expectations [52,53]. The reduction in saturated fat is essential for mitigating the risks of cardiovascular diseases and metabolic disorders, both of which are closely linked to high-fat diets [43]. Countless research efforts have demonstrated that incorporating oleogels into meat-based products can address these health concerns without compromising sensory qualities, which remain critical to consumer acceptance [37,39,43,52,53,54,55]. This balance between enhancing health benefits and maintaining product quality positions oleogels as a key innovation in the development of healthier meat products [6].

## 6. Optimized Method of Oleogel Preparation for Sausage Product Application

The distinct chemical composition and structure of various gelators require different strategies for forming oleogels, classified into direct and indirect technology. In direct methods, gelators are dispersed into heated oil, mixed, and cooled to form a network that solidifies the oil into a gel [56]. In contrast, indirect methods involve multi-step processes where the gelator is first dissolved in a secondary solvent, such as water or alcohol, before the solvent is removed through evaporation, drying, or other techniques. This leaves the gelator in the oil phase to form the oleogel. Indirect methods, including solvent exchange, emulsion-templating, and foam-templating, are particularly useful for hydrophilic polymers or gelators that do not dissolve easily in oil [57]. While these techniques provide better control over the microstructure of oleogels, they often require additional processing steps and specialized equipment, increasing complexity and costs. On the other hand, the direct-dispersion method is the most suitable for oleogel production in sausage applications due to its simplicity, scalability, and ease of integration into existing food manufacturing processes. It is especially advantageous for large-scale sausage production, where high-throughput processes demand a straightforward, cost-effective approach. Although high temperatures during processing may cause oil oxidation, this can be mitigated by using antioxidants, lowering processing temperatures, or reducing processing time. The simplicity, speed, and efficiency of the direct-dispersion method make it the superior choice for sausage production [58]. Table 1 highlighted the versatility in formulating direct-dispersion oleogels for sausage applications, showcasing a range of oils combined with specific gelators. These combinations yielded varied oil-to-gelator ratios, from 72:28 to 98:2, allowing for targeted adjustments in texture and fat content to meet desired nutritional and sensory qualities. Processing temperatures varied from 65 °C to 140 °C, indicating that certain gelators and oils required higher heat for full dissolution and stability. After processing, cooling and setting conditions were tailored to each oleogel, with some requiring room temperature while others necessitated precise cooling (e.g., 4 °C) for periods ranging from overnight to several days. These storage durations enhanced gel firmness and stability, ensuring that oleogels maintained a texture and mouthfeel comparable to traditional fats in sausages.

## 7. Incorporation of Direct-Dispersion Oleogels in Sausage Manufacturing

**Figure 1:**
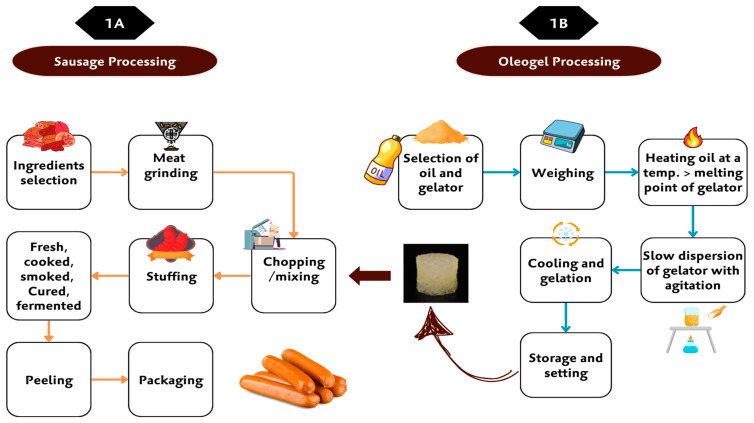
(**A**) Incorporation of oleogels during the mixing stage in sausage processing, (**B**) Preparation of oleogel for sausage application.

Sausage processing is a complex, multi-stage procedure designed to yield a product with desirable texture, flavor, and preservation qualities. Initially, ingredients are carefully prepared and measured to achieve a balanced composition. The primary components—meat and fat—are ground to achieve a specific particle size, facilitating a uniform texture and an ideal binding capacity, which are crucial for sausage structure. Seasonings, preservatives, and functional additives are then blended into the mixture to enhance flavor, prolong shelf-life, and stabilize the fat-protein matrix. This preparation creates a stable, uniform mix that can be further processed through cooking, smoking, curing, or fermentation, depending on the type of sausage being produced [9]. A critical point in sausage processing, particularly for modified formulations, is the mixing stage [58]. Here, oleogels are incorporated with other ingredients. These oleogels serve as a functional alternative to traditional fats, aiming to reduce saturated fat content while retaining essential textural and sensory qualities. When introduced at the mixing stage, oleogels are dispersed uniformly throughout the meat matrix. This uniform dispersion is essential not only for consistent fat replacement but also for ensuring that the oleogels contribute to a texture and mouthfeel similar to those provided by conventional animal fats. Figure 1A shows the incorporation of oleogels during the mixing steps in sausage production, while Figure 1B illustrates their preparation, demonstrating how oleogels can be integrated with other ingredients for uniform dispersion in the meat matrix, ensuring consistent fat replacement and desired product qualities.

## 8. Structuring Mechanisms of Oleogels and Their Roles in Sausage Applications

Oleogels contribute to sausage production through three primary structuring mechanisms: crystal particle systems, self-assembly systems, and polymeric network systems [36]. Each mechanism leverages specific gelators and structuring methods, creating diverse textures and qualities optimized for various sausage types. In crystal particle systems, low molecular weight gelators (LMWGs), such as beeswax, candelilla wax, and rice bran wax, dissolve in oil at high temperatures and crystallize as the mixture cools [73]. This crystallization process traps the oil within a structured network, emulating the texture and stability of animal fats like lard or tallow. This method is especially effective for imparting a firm texture, bite, and mouthfeel in emulsified meat products. The crystallized structure supports moisture retention, enhancing juiciness [74]. 

Self-assembly systems employ gelators such as phytosterols, sterols, and certain fatty acids, forming a three-dimensional network via weak, non-covalent interactions, such as hydrogen bonding and van der Waals forces [75]. Unlike crystal particle systems, these gelators create a softer, more flexible structure suited to sausages requiring a creamier texture, such as spreadable varieties. The network’s softness improves moisture retention and provides a smoother mouthfeel, making it ideal for fresh sausages with a softer bite [76].

Polymeric network systems use high molecular weight gelators (HMWGs), including ethyl cellulose, proteins, and polysaccharides. These gelators create stable networks through physical entanglement or chemical cross-linking, offering significant heat resistance [77]. This stability is ideal for cooked sausages, as it ensures the product retains its structure and consistency during cooking. The result is a firm bite and even fat distribution, essential for products like frankfurters and hot dogs, where fat reduction is achieved without compromising texture [78].

**Figure 2:**
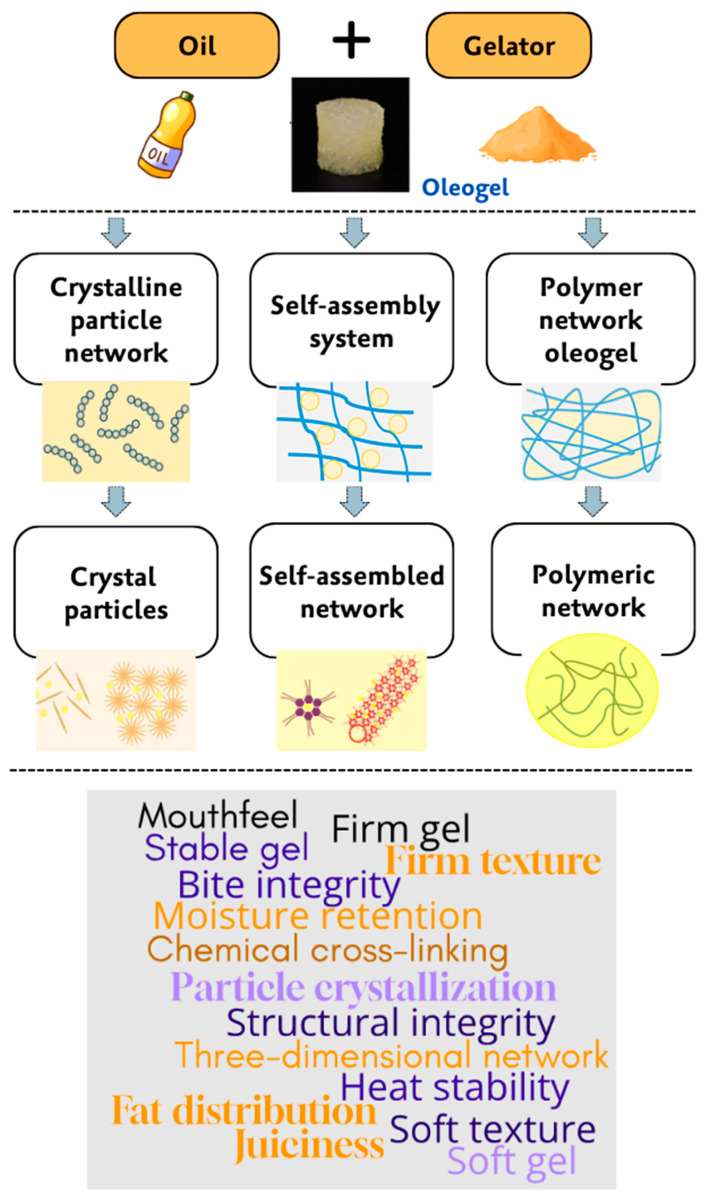
Oleogel mechanisms and word cloud of their formation and functions in sausage production.

Each oleogel structuring mechanism enhances sausage quality by replicating the texture and mouthfeel typically provided by animal fats while also improving moisture retention and thermal stability. These properties enable healthier sausage alternatives that preserve the essential sensory attributes of traditional formulations, adaptable to various sausage types and consumer preferences. Figure 2 illustrates the various oleogel mechanisms, accompanied by a word cloud highlighting oleogel formation and functions in sausage production.

## 9. Stability and Processing Dynamics in Oleogel-Enhanced Sausages

The incorporation of oleogels in sausage formulations presents a transformative approach to enhancing product stability and processing dynamics, crucial for maintaining structural integrity and optimizing production metrics. Oleogels, by their nature, could impact sausage emulsion stability, a critical factor in achieving a cohesive texture necessary for high-quality sausages [59]. This stability directly influences slice-ability and structural resilience, ensuring that sausages retain their shape and texture during handling, processing, and consumer use [64]. In terms of production outcomes, analyzing yield and loss parameters—specifically, total yield, fat retention, cooking losses, and overall weight stability—provides vital insights into oleogel’s role in operational efficiency and product uniformity [59,65,70]. These metrics indicate how well oleogels support the functional demands of sausage manufacturing. Ultimately, understanding and optimizing these parameters are essential tasks for creating oleogel-enhanced sausages that meet industry quality standards, offering a synergy of robust processing performance and product consistency.

### 9.1. Emulsion/Batter and Structural Stability

Incorporating oleogels into sausage formulations significantly enhances emulsion stability and slice-ability, offering structural advantages over traditional formulations without oleogels. This approach allows for the replacement of animal fats, maintaining product quality and texture. Tarté et al. (2020) demonstrated the effectiveness of high oleic and conventional soybean oil oleogels structured with rice bran wax in bologna sausages, noting that both 10% and 2.5% rice bran wax concentrations improved emulsion stability compared to samples with only liquid soybean oil. Notably, samples with 10% rice bran wax showed significantly less water and lipid separation, creating a stable matrix that reduced fluid release and reinforced cohesion, closely approximating the stability of pork backfat [59]. Similarly, Wolfer et al. (2018) observed that in frankfurter sausages, 2.5% and 10% rice bran wax oleogels with soybean oil improved emulsion stability, with 10% concentrations achieving superior moisture retention and structural integrity. This highlights the significance of utilizing higher gelator concentrations, such as 10%, to achieve stability, thereby establishing structured oleogels as viable alternatives to traditional animal fats in emulsified products [63].

Further, Ferro et al. (2021) explored 5% glyceryl monostearate-based oleogels with sunflower and high oleic sunflower oils in bologna sausages. Despite a lower gelator concentration, these oleogels achieved high emulsion stability and minimal liquid separation, proving effective as fat substitutes. Additionally, oleogels improved slice-ability; for example, sausages with 10% pork fat replaced by oleogel exhibited a higher percentage of intact slices than those with pork fat, indicating better structural integrity. Sausages with 20% pork fat replaced by oleogel showed even greater consistency, yielding more cohesive slices [64]. The enhanced emulsion stability and slice-ability are attributed to the compact network formed by oleogels, which strengthens product cohesion and stability, highlighting the potential of oleogels to serve as a balanced, functional fat replacement in sausage products [74].

### 9.2. Cooking Yield and Loss Analysis

Oleogels structured with rice bran wax concentrations in soybean oil, particularly at the 10% level, have demonstrated remarkable efficacy in preserving cooking yields in Bologna sausages. This effectiveness is largely due to their role in stabilizing both moisture and fat retention. The concentration of rice bran wax within these oleogels is pivotal; at the 10% gelator level, a robust three-dimensional network forms, featuring a higher melting point that withstands thermal breakdown during cooking [59,63]. This network serves as a physical matrix within the meat structure, efficiently entrapping fat and moisture. Consequently, oleogels mitigate the release of fat and curtail moisture loss, leading to a substantial reduction in cooking loss—a crucial determinant in yield retention for processed meats such as frankfurters and Bologna sausages. Unlike traditional fats and liquid oils, which lack the structural cohesion to prevent “cook out”, oleogels establish a semi-solid scaffold that anchors lipids [74].

Oleogels, crafted from a blend of flaxseed oil, sunflower wax, and beeswax at a 90:10 (*w*/*w*) ratio, have demonstrated considerable efficacy in reducing cooking loss in sucuk sausages compared to traditional formulations utilizing tallow fat [65]. This reduction is noteworthy as it suggests a higher retention of moisture and weight during cooking, thereby enhancing the juiciness and succulence of the final sausage product. The study’s findings reveal a distinct pattern in cooking loss across different fat formulations: sucuk sausages made with tallow fat exhibit the greatest cooking loss, while those incorporating beeswax and sunflower wax oleogels show a marked decrease in cooking loss. The mechanisms underlying this reduction in cooking loss with oleogels, as opposed to traditional fats, can be largely attributed to differences in melting points. Traditional fats, with relatively low melting points, tend to liquefy rapidly when exposed to heat, leading to significant fat drainage and a corresponding increase in cooking loss. Conversely, oleogels exhibit higher melting points, allowing them to maintain a semi-solid state for longer under heat, which aids in moisture preservation by reducing fat migration and leakage [65]. Further enhancing their effectiveness, the structural properties of oleogels play a key role in minimizing cooking loss. The oleogel matrix forms a stable network that effectively encapsulates moisture, limiting the evaporation of free water during cooking. This structural stability results in reduced cooking and weight loss, yielding a final product with improved moisture retention, better texture, and reduced fat loss throughout the cooking process [36]. Table 2 lists recent findings on the yield and processing losses associated with oleogel incorporation in sausage products.

## 10. Nutritional Modifications in Sausage Products with Oleogel

Exploring the potential of oleogels in sausage formulations opens up a promising approach for enhancing nutritional value while maintaining the desired sensory and texture qualities of sausage products. Oleogels, with their ability to modulate both fat and moisture levels, can significantly influence the texture and health profile of sausages. By replacing traditional fats with oleogels, formulations achieve a balance that helps retain moisture and, importantly, reduces overall fat content. This substitution offers a path toward healthier meat products without sacrificing quality [6]. The integration of oleogels as partial replacements for pork backfat in sausage production impacts moisture retention and fat content due to the unique structural and compositional properties of oleogels. By carefully controlling gelator-to-oil ratios, oleogels form a stable, semi-solid matrix that both immobilizes oil and retains water within the sausage, enhancing moisture levels [69]. A study on fermented sausages demonstrated that an olive oil-based oleogel with a monoglyceride ratio of 85:15 (*w*/*w*) as a fat substitute significantly affected moisture and fat retention. During fermentation, this oleogel matrix served as a moisture barrier, effectively slowing dehydration and leading to higher moisture content, which ranged from 41.49% to 47.28% depending on the specific formulation [60]. This retention likely results from the oleogel’s capacity to coat meat particles and create a barrier, preserving moisture during processing [55]. Furthermore, substituting pork backfat with olive oil oleogel was associated with a modest reduction in total fat content, particularly in formulations where 50% of the pork fat was replaced. This fat reduction demonstrates the oleogel’s potential for lowering lipid content while maintaining essential structural integrity in the sausage. Similarly, an investigation into Harbin red sausage formulations using peanut oil oleogels structured with ethyl cellulose revealed how varying ethyl cellulose concentrations, especially at 10%, enhanced stability and reduced oil loss. Replacing pork fat at levels from 10% to 50% with the peanut oil oleogels resulted in a significantly lower total fat content compared to the control. This reduction is attributed to the oleogel’s lower lipid density and high oil-binding capacity, which limit fat release during cooking [61]. The maintained moisture content, comparable to traditional sausage formulations, further underscores the oleogel’s ability to retain water, likely due to the stable three-dimensional network formed by ethyl cellulose. Oleogels also facilitate fat reduction by substituting part of the saturated animal fat with plant-derived oils, inherently lower in saturated fats and free from cholesterol [79]. In semi-smoked and red sausage formulations, replacing pork backfat with oleogels—ranging from 50% to 100%—led to significant decreases in total fat content, as plant oils in the oleogels contribute fewer lipids than animal fat [67]. This balance between moisture retention and fat reduction can be attributed to the oleogel’s gelled network, which not only limits fat release during thermal processes but also supports a more stable moisture environment. Consequently, oleogels provide a scientifically backed strategy for enhancing the nutritional profile of meat products by reducing fat content while preserving essential moisture, thereby improving both the health aspects and textural qualities of the final product.

Moreover, oleogels offer the possibility of a more beneficial fatty acid profile, favoring unsaturated fats that are associated with improved cardiovascular health [80]. The composition of the gelators used in oleogels—such as ethylcellulose, rice bran wax, and glyceryl monostearate—plays a key role in enhancing the fatty acid profile of meat products without contributing to higher levels of saturated fatty acids (SFAs) [81]. Unlike traditional animal fats, which are high in SFAs, these gelators themselves do not introduce additional SFAs into the formulation. Instead, they structure unsaturated vegetable oils, which are typically rich in monounsaturated (MUFAs) and polyunsaturated fatty acids (PUFAs), into a gel matrix, thereby preserving and enhancing the product’s nutritional profile [82,83]. For instance, in the reformulated Harbin red sausage, the replacement of pork back fat with peanut oil oleogels structured with ethylcellulose at 6–12% concentrations resulted in a reduction in SFA while increasing MUFA and PUFA. The ethylcellulose-based oleogel effectively gelled the peanut oil, which is naturally high in MUFAs, thereby reducing the overall SFA content of the sausage without compromising its texture and sensory qualities [61]. In frankfurters, the use of rice bran wax at 2.5% and 10% concentrations with soybean oil, replacing up to 50% of pork back fat, showed a significant decrease in SFA and a corresponding increase in PUFA and MUFA content. The rice bran wax oleogel improved the PUFA/SFA ratio of the sausage without introducing additional SFAs, as the rice bran wax serves as a structuring agent rather than a fat source. Data from the study indicated that PUFA content increased substantially in frankfurters with 10% rice bran wax oleogel, and the reduction in SFA contributed to a more heart-healthy fatty acid profile [63]. In Bologna sausages, oleogels using glyceryl monostearate (5%) with sunflower oil or high oleic sunflower oil replaced pork fat by up to 100%, further enhancing the PUFA and MUFA levels while reducing SFAs. Sunflower oil, particularly high oleic variants, is rich in MUFAs and PUFAs, which are retained within the gel matrix formed by glyceryl monostearate. As a result, the Bologna sausage exhibited a PUFA/SFA ratio that was markedly improved, with the data showing higher MUFA and PUFA levels than the control samples. For example, sausages with 100% replacement showed an increase in PUFAs while maintaining low SFA levels, thanks to the properties of glyceryl monostearate, which does not add saturated fats but enables the structuring of healthier oils. The low SFA contribution of these gelators and their ability to stabilize unsaturated oils in a gel matrix ensure that the oleogels contribute to a more favorable fatty acid profile [64]. By preventing the oils from undergoing rapid oxidation and by encapsulating them effectively, the gelators preserve the nutritional benefits of high-MUFA and high-PUFA oils, making them valuable alternatives for producing meat products with healthier lipid profiles. Table 2 summarizes recent studies on the effects of oleogel on fat content and the enhancement of the fatty acid profile in sausage formulations.

Additionally, by integrating oleogels, there is potential to lower cholesterol content in sausages, aligning these products with current nutritional guidelines. The influence of oleogels on cholesterol reduction in meat products is closely linked to their ability to replace traditional animal fats. For instance, in Thai sweet sausage, rice bran wax/rice bran oil (2:98) oleogels (RBOG) were used as partial fat replacers, substituting pork backfat at levels of 0%, 25%, 50%, and 75%. By replacing animal fats with RBOG, which inherently lacks cholesterol, these formulations reduce overall cholesterol levels, providing a healthier alternative without compromising the fat-binding properties essential for texture and moisture retention [71]. The rice bran wax and oil structure also promote a stable gel matrix that supports these benefits, showing that oleogels are not only functional fat substitutes but also contribute positively to the nutritional profile by lowering cholesterol content in final products.

## 11. Effects of Oleogel on Texture and Sensory Properties in Sausages

For the successful adoption of oleogel-enriched sausages, it is imperative to achieve specific quality attributes, including texture and sensory properties. Texture and microstructure play a foundational role in emulating the firm, succulent bite characteristic of traditional sausages, with oleogels providing structural integrity and moisture retention necessary for consumer satisfaction. Equally vital are the sensory properties—taste, aroma, and mouthfeel—that oleogels must effectively replicate to deliver an experience akin to that of conventional animal fats, ensuring a rich texture and pleasant flavor release. These integrated properties collectively enhance the likelihood of oleogel-enriched sausages meeting both consumer acceptance criteria and rigorous industry standards, positioning them as a viable, health-conscious alternative in the market.

### 11.1. Instrumental Texture Profile Analysis and Microstructure

Incorporating oleogels into sausage formulations has yielded varied effects on textural properties, influenced by factors such as the meat matrix, type of oil, and selected gelator. While some studies indicate minimal impact on texture, others reveal that oleogels affect textural values. Importantly, none of the research reviewed reports significant or adverse deviations from control samples, underscoring the compatibility of oleogels with traditional sausage textures and highlighting their potential as adaptable components in healthier sausage products without compromising sensory quality. For instance, Tarté and colleagues (2020) demonstrated that oleogels made with 90.0% or 97.5% of either conventional or high oleic soybean oil and rice bran wax as a gelator could replace up to 41.9% of pork backfat in bologna sausage without significantly affecting texture. The rice bran wax gelator, applied at 2.5% and 10% of the oil content, effectively replicated the firmness and chewiness of pork fat by forming a stable matrix that maintained key texture profile parameters and incisor peak force, thus mimicking the solid fat structure [59]. Similar results were observed in frankfurter-type sausages, where rice bran wax/soybean oil oleogels (2.5:97.5 and 10:90) replicated the firmness, chewiness, and springiness of traditional pork fat [63]. In contrast, Ferro (2021) reported that monoglyceride-based oleogels (5%) made from conventional and high-oleic sunflower oils, used to replace varying amounts of pork fat (25%, 50%, 75%, and 100%) in Bologna sausages, influenced texture by increasing hardness as the proportion of pork fat replacement increased. This effect was attributed to the compact structure formed with higher oleogel content, affecting the sausage’s slice-ability and resulting in a higher slice count compared to the control. These textural changes are likely due to the stable gel-like properties (G′ > G″) of monostearate-based oleogels, which partially mimic pork fat’s structure while enhancing unsaturated fatty acid content [64]. Similar findings by Shao (2023) noted that replacing 10–30% of pork fat with peanut oil oleogels structured with 6–12% ethylcellulose led to increased hardness and structural stability in Harbin red sausages, indicated by higher storage (G′) and loss (G″) moduli [61]. On the other hand, a different study structured sunflower oil with a 15:5 weight ratio of monoglycerides to phytosterols, replacing 50% of pork backfat in frankfurter sausages. The oleogel-substituted sausages showed lower values in hardness, brittleness, gumminess, and chewiness compared to controls with full pork backfat, likely due to structural differences in crystalline properties between the oleogel and pork fat [62]. Table 2 presents recent research on the influence of oleogel on texture characteristics in sausage formulations.

**Figure 3:**
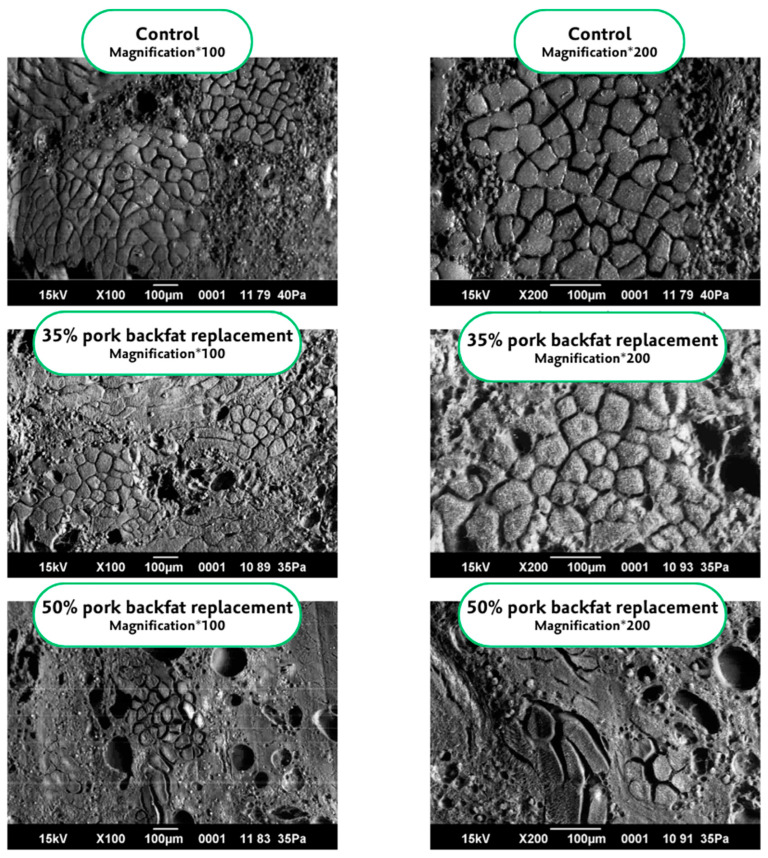
Microstructure images of semi-smoked sausages with sunflower oil oleogels structured using a 2:1 monoglyceride-beeswax blend at 7% and 10% concentrations, replacing 35% and 50% pork backfat, respectively, showing the control sample with notable porosity and large lipid granules, while increased oleogel content resulted in a more homogeneous structure with reduced granules, with the 7% oleogel at 35% replacement closely resembling the control structure and exhibiting improved uniformity. Reproduced from [67] with permission.

Regarding microstructure analysis, sausages containing oleogels revealed distinct differences compared to traditional pork fat formulations. In a recent study, control samples with pork fat exhibited the largest fat globules, with 49.9% of globules exceeding 100 μm^2^, significantly more than in samples containing rice bran wax oleogels. In oleogel-treated samples, the structure appeared more compact, with fewer visible fat globules compared to pork fat and soybean oil samples. Specifically, image analysis indicated that 14.46% of the pork fat sample area consisted of lipids, a marked contrast to 2.65% in 2.5% rice bran wax and 6.88% in 10% rice bran wax oleogel treatments. This suggests that the oleogel structure may have fragmented into smaller particles, undetectable by imaging software, or dissolved into the protein phase due to the hydrophilic properties of certain rice bran wax components [63]. Scanning electron microscopy in another study further supported these findings, showing that reduced pork fat levels led to a denser, more compact sausage structure. Control formulations with 20% and 10% pork fat displayed a uniform and porous structure typical of stable meat emulsions, with more pores observed in 10% pork fat, likely due to the higher water content from lower fat. In contrast, formulations with 100% oleogel replacement exhibited a significantly denser network, correlating with increased hardness and higher G′ values in the meat batters, indicating a firmer structure likely due to the oleogel’s integration into the protein-fat matrix [64]. These findings imply that partial oleogel replacement effectively preserves traditional emulsion structures, while full replacement might alter microstructural characteristics, increasing density and firmness. Figure 3 illustrates the microstructural properties of sunflower oil oleogels structured with a 2:1 monoglyceride-beeswax blend at 7% and 10% concentrations, and their effects on semi-smoked sausages.

The textural effects of oleogel incorporation in sausage formulations depend on key variables: the oil-to-gelator ratio, fat replacement level, oleogel type, and processing method. Higher oil-to-gelator ratios generally yield a more structured texture, while lower ratios contribute to softer textures. Partial fat replacements often preserve traditional textures more effectively, whereas complete replacements may lead to a firmer product due to increased compactness. The oleogel type influences the replication of conventional fat’s structural attributes, particularly affecting chewiness and cohesiveness. Additionally, the processing method, including heat application and mechanical handling, modifies oleogel behavior within the sausage matrix, necessitating tailored adjustments for optimal textural outcomes. Exploring each of these factors relative to specific sausage types will be crucial in developing oleogel-based formulations that align with targeted textural expectations.

### 11.2. Sensory Performance

Sensory analysis plays a pivotal role in food product development and optimization, providing insights into consumer acceptance through attributes such as taste, texture, aroma, color, overall mouthfeel, and acceptability. In the context of sausage formulations utilizing oleogels as a fat substitute, sensory evaluations yield mixed results, underscoring the delicate balance between health benefits and desirable sensory qualities. While some formulations have successfully retained textural properties similar to traditional fat-based sausages, achieving a familiar mouthfeel and taste profile, others faced rejection due to noticeable deviations in texture or flavor from conventional sausage expectations. For instance, Igenbayev (2023) explored sausage formulations where pork fat was partially replaced with beeswax-structured oleogels. Two experimental samples were developed, containing 7% and 10% oleogel, respectively, using a formulation of sunflower oil structured with monoglyceride and beeswax in a 2:1 ratio to ensure texture and stability. Sensory analysis revealed that both oleogel samples exhibited a uniform texture, appealing red-pink color, and the absence of gray spots, along with a pleasant aroma and semi-smoked sausage taste. However, while the 7% oleogel sample maintained favorable sensory qualities, the 10% inclusion resulted in significant declines in texture and juiciness. Sensory panelists rated the 10% sample lowest for appearance and cross-sectional quality, suggesting that higher oleogel levels might negatively impact consumer acceptability due to textural and juiciness losses [67].

In another investigation, sensory analysis of sucuk sausages incorporating oleogels (Flaxseed oil-sunflower wax/beeswax) compared to a traditional tallow-based control provided valuable insights. Panelists evaluated samples across seven sensory descriptors: appearance, hardness, chewiness, fattiness, juiciness, aroma, and flavor. The control sample scored highest in appearance, followed by samples containing sunflower wax oleogel and beeswax oleogel. The control also ranked significantly firmer in sensory hardness, aligning with measurements that confirmed oleogels’ limitations in replicating tallow fat’s hardness. Conversely, oleogel samples were perceived as chewier due to oleogels’ softer consistency at mouth temperature, producing a tender, chewable texture. Interestingly, although all samples had the same fat content, oleogel-enriched samples scored highest in fattiness perception, possibly enhanced by beeswax oleogel. Differences in juiciness were also notable, with beeswax oleogel samples ranking highest, followed by sunflower wax and the control. This variation may result from higher melting points of beeswax and sunflower wax oleogels, which retain moisture during cooking. However, aroma and flavor ratings favored the control sample, highlighting that while oleogel replacements provide nutritional benefits, they fall short of delivering the rich sensory qualities of traditional fat-based sucuk [65].

Franco (2019) assessed sensory preferences when pork backfat was partially substituted with linseed oleogel, composed of linseed oil and beeswax (8% *w*/*w*). The control sample was favored in appearance, particularly regarding color, as it exhibited lower yellowness, which often correlates with better consumer acceptance [84,85,86]. Higher yellowness in meat products is generally linked to rancidity from lipid oxidation, reducing consumer appeal. Significant differences were also observed in odor and taste, with the control sample rated most favorably. These findings indicate that while linseed oleogel can replace pork fat, higher substitution levels may compromise consumer acceptability due to altered appearance, odor, taste, and juiciness [52]. These differences often stem from the specific types and concentrations of oleogels used, as well as their interactions with other ingredients, which influence characteristics such as firmness, juiciness, and cohesiveness. This feedback is essential for refining oleogel formulations, highlighting the need to optimize oleogel attributes to closely mimic traditional pork fat’s sensory qualities while offering a healthier alternative. Table 2 lists recent findings on the sensory properties of sausages with oleogel incorporation.

## 12. Effect of Oleogels on Oxidative Stability in Sausage Products

The application of oleogels as partial fat replacements in sausage formulations has shown promising results in managing lipid oxidation while offering healthier alternatives to traditional animal fats. In Harbin red sausage, ethylcellulose oleogels with peanut oil at concentrations of 6–12% were used to replace pork fat at levels ranging from 10–50%. Results indicated that lower replacement levels (10–30%) maintained oxidative stability similar to the pork fat control, making these levels optimal for reducing saturated fat without compromising product oxidative stability. However, higher replacement levels (40–50%) led to increased lipid oxidation due to the unsaturated nature of peanut oil [61]. In frankfurter sausages, a sunflower oil oleogel structured with a 15:5 monoglycerides-to-phytosterols ratio replaced 50% of pork backfat, achieving comparable oxidative stability to the control over 40 days. The interaction of monoglycerides and phytosterols formed a robust network structure that limited oxidation effectively, supporting its suitability for fat replacement in processed meats [62]. Additionally, in another frankfurter study, rice bran wax oleogels with soybean oil at 2.5% and 10% concentrations were used to replace pork fat. While the 2.5% rice bran wax oleogel maintained oxidation levels close to the pork fat control, the 10% rice bran wax showed higher Thio-barbituric acid values, likely due to oxidation occurring from prolonged heating during oleogel formation. Nevertheless, both concentrations kept lipid oxidation within acceptable limits throughout storage, with lower concentrations proving particularly effective in balancing fat reduction and oxidation control [63]. Collectively, these studies suggest that oleogels structured with specific gelators and used in controlled concentrations—typically up to 30% replacement—can serve as viable fat substitutes in various sausage types, promoting healthier profiles while managing lipid oxidation effectively. Table 2 details recent studies on the oxidative stability of sausage products enhanced with oleogel incorporation.

## 13. Conclusions and Future Perspectives

This review highlights the potential of oleogels developed using the direct-dispersion approach as a compelling alternative to conventional fats in sausage production, emphasizing crucial quality considerations for integrating oleogels into sausage formulations. A diverse range of gelators, encompassing both low and high molecular weight compounds, can be utilized to structure various plant-based oils. The selection of the oil/gelator type and the corresponding ratios significantly influences the functional properties of sausages. Different gelators can elicit distinct effects on the texture, stability, and overall quality of the product. Furthermore, the choice of oil, particularly those rich in favorable fatty acids, can enhance the nutritional profile of the sausage, contributing to improved health outcomes by reducing saturated fat levels and increasing the content of unsaturated, health-promoting fatty acids. Oleogels have the potential to address the rising consumer demand for healthier meat products without compromising quality. Despite these advantages, a key challenge lies in refining oleogel formulations to closely replicate the sensory and texture characteristics of animal fats. Advancements in structuring techniques and sensory optimization could further enhance oleogels’ suitability for commercial sausage production. Scaling the application of oleogels in industrial sausage production will require careful optimization of formulation, processing, and equipment to maintain consistent quality and functionality at larger scales. At the industrial level, precise blending is crucial to ensure uniform distribution of oleogels in the sausage matrix while preserving the gel’s structure under varying temperature conditions. Additionally, rigorous quality control measures will be necessary to monitor texture, shelf-life, and sensory characteristics, ensuring the final product aligns with consumer expectations. Key considerations for commercial success include cost efficiency, regulatory compliance, and proper labeling, particularly as health-conscious consumers increasingly demand alternatives to saturated fats in processed foods. Future research should prioritize enhancing oleogel formulations for greater stability, scalability, and sensory appeal, thus supporting broader industry adoption and consumer acceptance. This progress will not only help meet public health goals but also provide nutritionally improved meat products without compromising on quality or appeal.

## Figures and Tables

**Table 1:** Development of various direct-dispersion oleogels for sausage applications.

Oil|Gelator	Oil:Gelator	Processing Temp.|Time	Setting Conditions	Ref.
Conventional soybean oil, High oleic soybean oil|Rice Bran Wax	90:10 and 97.5:2.5	90 °C|2 h	Set for 7 days at ambient temperature	[59]
Olive oil|MG95 (Monoglycerides)	85:15	90–95 °C|60 min	Cooled for 40 min at ambient temperature, stored at 5 °C for 3 days	[60]
Sunflower oil, Peanut oil, Corn oil, Flaxseed oil|Ethyl Cellulose	94:6, 92:8, 90:10, and 88:12	140 °C|Until dissolved	Cooled to room temperature, stored at 4 °C	[61]
Sunflower oil|Monoglycerides and Phytosterols	80:20	90 °C|Up to 60 min	Cooled and stored at 18–20 °C	[62]
Conventional soybean oil|Rice Bran Wax	90:10 and 97.5:2.5	90 °C|2 h	Stored at 2.7 °C for 5–7 days	[63]
High oleic sunflower oil, Sunflower oil | Glyceryl Monostearate	95:5	90 °C|15 min	Cooled to room temperature, stored for 48 h at 25 °C, then 24 h at 4 °C	[64]
Flaxseed oil|Sunflower Wax, Beeswax	90:10	80 °C|Until dissolved	Cooled to ambient temperature (23 ± 2 °C) overnight	[65]
Canola oil|Candelilla Wax	97.5:2.5	100 °C|20 min	Stored for 5 days at 4 °C	[66]
Sunflower oil|Monoglycerides and Beeswax	80:20	90–95 °C|60 min	Cooled and stored at 4 °C for 24 h	[67]
Linseed oil|Beeswax	92:8	80 °C|30 min	Cooled at ambient temperature until gel formation	[52]
Olive oil-chia oil|Beeswax	90:10	65 °C|Until dissolved	Cooled for 60 min at room temperature, then stored at 3 °C	[68]
Linseed oil|Beeswax, γ-Oryzanol, and β-Sitosterol	92:8	80 °C|Until dissolved	Stored at ambient temperature	[69]
Sunflower oil|Candelilla Wax, Glyceryl Monostearate	90:10	80 °C|Until dissolved	Cooled at 4 °C for 24 h before use	[70]
Rice bran oil|Rice Bran Wax	98:2	80 °C|Until dissolved	Cooled at room temperature and left for overnight	[71]
Canola oil|Lecithin, Sorbitan Monostearate	72:28	80 °C|30 min	Cooled at 25 °C for 24 h, stored at 4 °C for 2 days	[72]

**Table 2:** Effects of direct-dispersion oleogels on sausage properties with varying levels of pork backfat replacement.

Oleogel Matrix	Sausage Type	%Fat Replacement	Sausage Properties						Ref.
			Yield And Losses	Fat Content	Fatty Acid Profile	Texture	Sensory	Oxidative Stability	
Conventional soybean oil, High oleic soybean oil|Rice Bran Wax	Bologna	41.9% of pork backfat	No yield differences in oleogel-treated and control samples	Slightly lower fat content in CSO-oleogel-treated samples than control	Linoleic & α-linolenic acids were highest in CSO-oleogel-treated samples, lower in HOSO-oleogel-treated samples, lowest in control. Oleic ↓ in CSO, ↑ in HOSO vs. Control	Lipid source and storage time had no significant effects on texture profile or incisor peak force	No differences were found in aroma, flavor, texture, or moistness between control and oleogel-treated samples	TBARS values stayed consistently low over the 98-day storage period	[59]
Olive oil|MG95 (Monoglycerides)	Fermented	50% pork backfat	Lower weight loss after cooking in oleogel-treated samples than control	Fat content reduced by 6.72% in oleogel-treated samples	SFA reduced by 17.4%, MUFA increased by 9.4%, and cholesterol reduced by 18.8% in oleogel-treated samples compared to control	ND	Oleogel samples ranked second after control samples	ND	[60]
Peanut oil|Ethyl Cellulose	Harbin red sausage	10–50% pork backfat	ND	All oleogel-treated samples showed a significant ↓ in total fat, with the lowest fat content in 50% treatment	In oleogel-treated samples, SFAs dropped from 39.9% to 28.4%, while UFAs rose from 55.7% to 68.4%. ω-6 FAs increased by 23–122%, but ω-3 FAs showed a slight ↓ vs. pork fat control	No significant influence in oleogel-treated samples compared to control	No significant influence in oleogel-treated samples compared to control	Higher lipid oxidation in oleogel-treated samples due to greater degree of unsaturation of peanut oil	[61]
Sunflower oil|Monoglycerides and Phytosterols	Frankfurter	50% pork backfat	ND	ND	ND	The control samples showed higher hardness, brittleness, gumminess, and chewiness than oleogel-treated samples, while cohesiveness and elasticity showed no difference	No differences were found	No differences were found	[62]
Conventional soybean oil|Rice Bran Wax	Frankfurter	80.65% pork backfat	No differences were found in cooking yield	No differences were found	Oleogel-treated samples had higher PUFAs (18:2n6, 18:3n3) and lower SFAs (18:0, 16:0) and MUFA (18:1c9) than control samples	Oleogel-treated samples were similar to pork fat treatment in firmness, chewiness, and springiness	Pork fat replacement did not affect aroma but significantly reduced flavor	Higher lipid oxidation in oleogel-treated samples due to higher degree of unsaturation in soybean oil	[63]
High oleic sunflower oil, Sunflower oil|Glyceryl Monostearate	Bologna	25, 50, 75 and 100% of pork backfat	ND	50% replacement samples showed a significant fat reduction (~25%) compared to the control	SFA was reduced by up to 67% in oleogel-treated samples, while MUFA (high oleic oleogels) and PUFA (sunflower oleogels) increased	Hardness increased in the oleogel-treated samples compared to control	Both oleogel-treated and control samples had good consumer acceptance, with no significant differences	ND	[64]
Canola oil|Candelilla Wax	Frankfurter	50–100% of pork backfat	ND	Higher contents of fat in oleogel-treated samples	ND	Sausages with 50% fat replacement had lower hardness, while 100% oleogel samples showed higher hardness. Adhesiveness, cohesiveness, elasticity, and resilience remained unchanged	ND	ND	[66]
Sunflower oil|Monoglycerides and Beeswax	Semi-Smoked	7% and 10% of pork backfat	ND	No differences were found	SFA decreased by 35–38%, while MUFA and PUFA increased	ND	No differences were found	ND	[67]
Linseed oil|Beeswax	Frankfurter	25–10% of pork backfat	ND	Higher contents of fat in oleogel-treated samples	SFA reduced from 35.15 g/100 g to 33.95 g and 32.34 g/100 g in 25% and 50% replacement samples, achieving more balanced n-6/n-3 ratios, compared to control	Cohesiveness, gumminess, and chewiness slightly increased in oleogel-treated samples compared to control sausages	Control had better appearance, lower yellowness (less rancidity), and higher scores in odor, taste, and juiciness	ND	[52]
Linseed oil|Beeswax, γ-Oryzanol, and β-Sitosterol	Fermented	20% and 40% of pork backfat	ND	Fat content ranged from 33.01% to 39.19%, lower than in traditional sausages	The fatty acid profile improved nutritionally, with PUFA/SFA and n-6/n-3 ratios of 1.41 and 0.93 in oleogel-treated samples	Beeswax oleogel resulted in notable changes in hardness, cohesiveness, gumminess, and chewiness, with sausages containing beeswax oleogel being less hard than control	Control was most accepted, while 20% replacements scored positively on the hedonic scale, and 40% replacements scored negatively	ND	[69]
Sunflower oil|CW, Glyceryl Monostearate	Bologna	100% of pork backfat	100% fat replacement raised fat loss, highest in GMS (7.75%) and CW (5.81%) oleogels	ND	ND	Control samples had higher hardness compared to oleogel-treated samples. Adhesiveness, cohesiveness, springiness, and gumminess were similar across samples	ND	ND	[70]

ND; Not determined, ↓; Decrease, ↑; Increase.

## Data Availability

No new data were created or analyzed in this study.
